# Association between red blood cell folate and *Trichomonas vaginalis* infection among women

**DOI:** 10.1186/s12879-022-07950-x

**Published:** 2023-01-23

**Authors:** Wan-Zhe Liao, Zhi-Yi Zhou, Jun-Hao Mao, Zi-Xun Wang, Yi-Ming Hu, Yong-Fu Lou, Qiao-Rui Zheng, Xu-Guang Guo

**Affiliations:** 1 Department of Clinical Laboratory Medicine, The Third Affiliated Hospital of Guangzhou Medical University, Guangzhou, 510150 China; 2 Department of Clinical Medicine, The Nanshan College of Guangzhou Medical University, Guangzhou, 511436 China; 3 Department of Clinical Medicine, The Third Clinical School of Guangzhou Medical University, Guangzhou, 511436 China; 4 Department of Computer Science, Nanjing University of Posts and Telecommunications, Nanjing, 210023 China; 5 Department of Electronic Information Engineering, Glasgow College, University of Electronic Science and Technology, Chengdu, 611731 China; 6 Guangdong Provincial Key Laboratory of Major Obstetric Diseases, The Third Affiliated Hospital of Guangzhou Medical University, Guangzhou, 510150 China; 7 Guangzhou Key Laboratory for Clinical Rapid Diagnosis and Early Warning of Infectious Diseases, KingMed School of Laboratory Medicine, Guangzhou Medical University, Guangzhou, China; 8 Department of Thoracic Surgery, Shangrao People’s Hospital, Shangrao, China

**Keywords:** *Trichomonas vaginalis*, RBC folate, NHANES, Multivariate regression models, Gynecological disease

## Abstract

**Background:**

Increased folic acid has been found to be latently protective against gynecological infection, including several kinds of vaginosis. In this study, we laid emphasis on whether RBC (Red Blood Cell) folate was associated with the infectious ratio of *Trichomonas vaginalis*, a kind of anaerobic parasitic protozoan.

**Methods:**

We set RBC folate as the exposure variable and *Trichomonas vaginalis* as the outcome variable. Other subsidiary variables were regarded as covariates that may work as potential effect modifiers. The cross-sectional study was conducted with two merged waves of the National Health and Nutrition Examination Survey (NHANES) from 2001 to 2004, and a sample of 1274 eligible women (1212 negative and 62 positive in *Trichomonas vaginalis* infection) was integrated for the exploration of the association between RBC folate and *Trichomonas vaginalis* infection. Multivariate regression analyses, subgroup analyses, and subsequent smooth curve fittings were conducted to estimate the relationship between RBC folate and *Trichomonas vaginalis* in women.

**Results:**

In the multivariable logistic regression analyses, a negative association was observed between stratified RBC folate status and *Trichomonas vaginalis* infection with all confounders adjusted. Referencing the lowest RBC folate concentration quartile, the higher concentration quartiles reported a relatively lower infection ratio, while there was a weak correlation between total RBC folate concentration and *T. vaginalis* (*Trichomonas vaginalis*) infection. In subgroup analyses stratified by BMI and age, this association was only found significant in high age and BMI groups.

**Conclusions:**

The cross-sectional study indicated a negative association between RBC folic acid and *Trichomonas vaginalis* infection, and latent effects of BMI and age on the association were also found.

**Supplementary Information:**

The online version contains supplementary material available at 10.1186/s12879-022-07950-x.

## Background

The pathology of *Trichomoniasis* is due to the damage to host epithelial cells by various processes during the infection, and its clinical manifestations also vary between men and women. 50 percent of women infected with *Trichomonas vaginalis* develop symptoms, and about 30 percent of asymptomatic cases develop itching and pain during sexual intercourse and secretion with foam 6 months after infection, while male infected people are generally asymptomatic, but they have mild urethritis, epididymitis and prostatitis [1, 2]. According to the World Health Organization (WHO), the global prevalence of Trichomoniasis in women is estimated at 5.3%, there were 156 million new cases of trichomoniasis in 2016 [3, 4], suggesting that *Trichomonas vaginalis* infection is a prevalent sexually transmitted disease [5]. It should be noted that asymptomatic *Trichomonas vaginalis* infection can be frequently found in women, causing misjudgment of its prevalence, which could interrupt the accurate diagnosis procedure [6]. Recent studies revealed associations between *Trichomonas vaginalis* infection and cervical cancer, pelvic inflammatory disease, adverse pregnancy, infertility, and transmission of HIV-1 [3, 7–9], making it a public health concern to investigate factors influencing *T. vaginalis* infection.

Folate is an essential trace element in the human body. It is involved in various metabolic activities and plays a vital role in the process of DNA methylation, biological metabolic transformation, cell proliferation, and differentiation [10]. Folic acid is a water-soluble vitamin, but humans cannot synthesize it alone. The concentration of folate in red blood cells (RBC) is an indicator of the body’s long-term folate status. Therefore, the major objective was to collect and analyze relevant data from the NHANES survey (2001–2004) among women of all races and explore the potential association.

## Methods

### Study population

The National Health and Nutrition Examination Survey (NHANES) is a program of studies designed to assess the health and nutritional status of adults and children in the United States and was conducted by the National Center of Health Statistics (NCHS) at the Centers for Disease Control and Prevention (CDC), providing us with a wealth of information about the nutrition and health of Americans through the use of multiple phases, probability sampling design and coding variables with the information collected concerning health and nutrition-related domains [11].

**Fig. 1:**
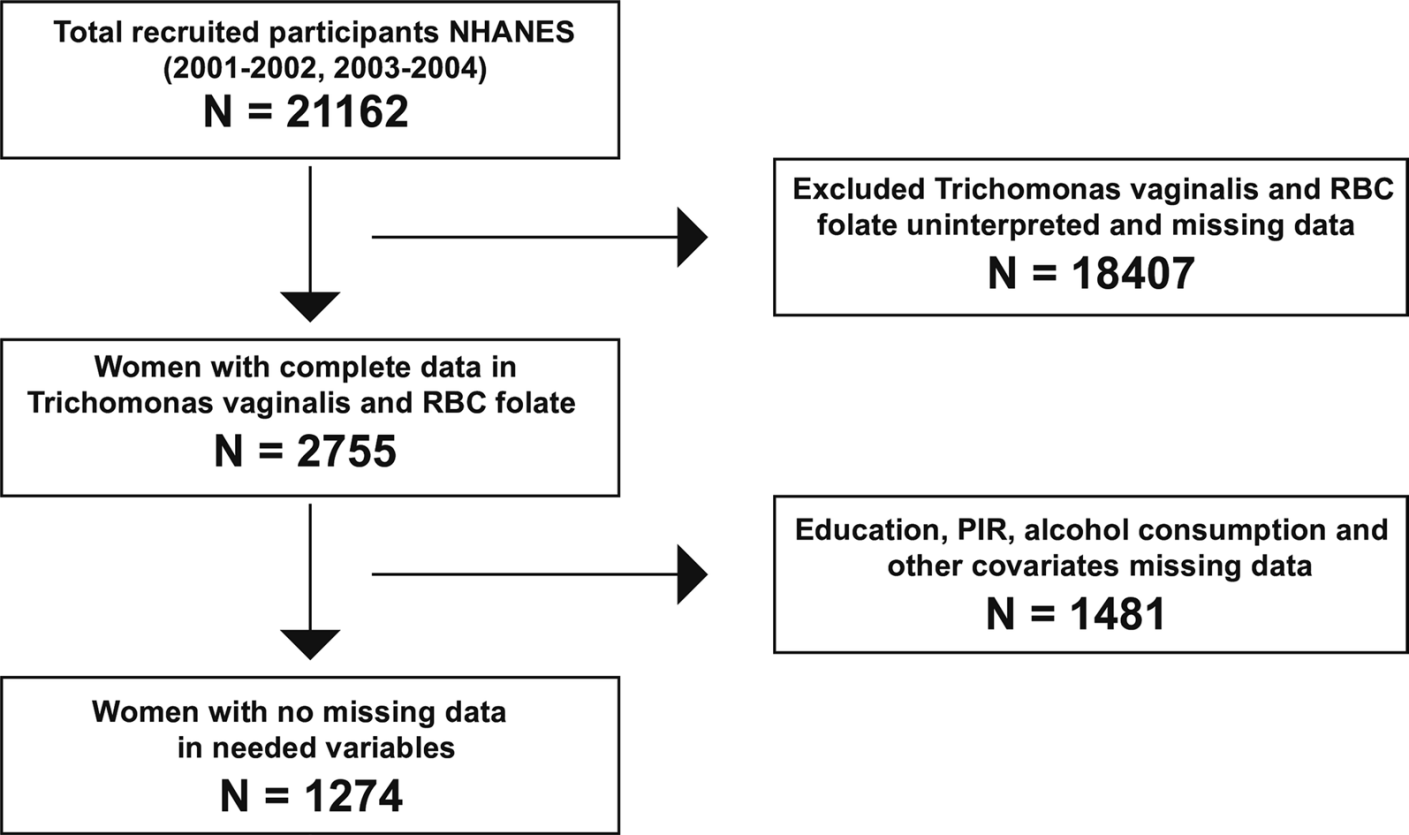
Flow chart of participant enrollment. Flow chart of participant enrollment for the analysis from the National and Nutrition Examination Survey 2001–2004 dataset

On the basis that the outcome variable, *T. vaginalis* infection was only coded in two circles of NHANES, we chose to merge the two circles, 2001–2002 and 2003–2004, and then conducted subsequent research. The flow chart for the inclusion and exclusion is shown in Fig. 1. With *T. vaginalis* infection and RBC folate uninterpreted and missing data (n = 18,407) were first excluded, then other variables including educational status, PIR (Poverty Income Ratio), alcohol use status, serum or plasma cholesterol, calcium dietary intake, BMI, and alcohol consumption missing data (n = 1481) dislodged, 1274 eligible women left were included in our analysis, whose baseline characteristics are shown in Table 1. The raw data and the filtered data were attached as Additional file 1 and 2.

**Table 1:** Characteristics of 1274 women overall and by *T. vaginalis* infection

	*Trichomonas vaginalis* infection			P-value
	Overall N = 1274	Negative N = 1212	Positive N = 62	
RBC folate (ng/ml), mean ± SD	278.19 ± 111.39	281.34 ± 111.84	216.74 ± 81.21	< 0.001
Age (years), mean ± SD	33.93 ± 8.55	33.78 ± 8.52	36.94 ± 8.62	0.004
PIR, median (IQR)	2.68 (1.29–4.61)	2.78 (1.34–4.64)	1.44 (0.62–2.46)	< 0.001
Dietary intake calcium, median (IQR)	700.00 (448.00–1060.75)	716.00 (457.00–1076.00)	447.00 (307.75–708.25)	0.002
BMI (kg/m^2^), mean ± SD	27.87 ± 7.02	27.71 ± 6.98	31.01 ± 7.04	< 0.001
Serum or plasma cholesterol, mean ± SD	5.08 ± 1.11	5.08 ± 1.12	5.03 ± 0.88	0.758
Race/ethnicity				0.162
Mexican American	246 (19.31%)	239 (19.72%)	7 (11.29%)	
Other Hispanic	48 (3.77%)	47 (3.88%)	1 (1.61%)	
Non-Hispanic Black and White	932 (73.16%)	879 (72.52%)	53 (85.48%)	
Other Race–including multi-racial	48 (3.77%)	47 (3.88%)	1 (1.61%)	
Marital status				< 0.001
Married/living with partner	805 (63.19%)	783 (64.60%)	22 (35.48%)	
Widowed/divorced/separated	168 (13.19%)	148 (12.21%)	20 (32.26%)	
Never married	301 (23.63%)	281 (23.18%)	20 (32.26%)	
Education				< 0.001
Less than high school	228 (17.90%)	206 (17.00%)	22 (35.48%)	
College	278 (21.82%)	259 (21.37%)	19 (30.65%)	
More than college	768 (60.28%)	747 (61.63%)	21 (33.87%)	
Alcohol consumption (drinks per day)	2.34 ± 1.73	2.31 ± 1.67	2.95 ± 2.54	0.005
Alcohol use status				0.194
Non-drinker (less than 2 drinks per day)	457 (35.87%)	439 (36.22%)	18 (29.03%)	
Moderate alcohol use (2 drinks per day)	414 (32.50%)	396 (32.67%)	18 (29.03%)	
High alcohol use (more than 2 drinks per day)	403 (31.63%)	377 (31.11%)	26 (41.94%)	

Mean ± SD or median (IQR): P values were calculated by one-way ANOVA (normal distribution) and Kruskal‒Wallis H (skewed distribution) test % for categorical variables. P values were calculated by the chi-square test

### Variables and covariates

The exposure variable was red blood cell folate, and the outcome variable was *T. vaginalis* infection. Erythrocyte folate was measured by collecting samples that were sent to the CDC’s National Centre for Environmental Health (NCEH) for analysis using a microbiological assay [12]. The samples were first diluted 1:11 with a solution of 1 g/dL ascorbic acid in water and either incubated for 90 min prior to assay or frozen immediately for later assay. The 90-min incubation or the freeze–thaw is necessary for hemolysis of the red blood cells, either allows the endogenous folate conjugates to hydrolyze the conjugated pteryl polyglutamates prior to assay. The sample is further diluted 1:2 with a protein diluent (human serum albumin), resulting in a matrix similar to that of the standards and serum samples. *T. vaginalis* infectious status was detected by performing PCR with primers from a region of the 18S rRNA gene that produce a 312 base pair product. By using hybridization, product specificity was validated with digoxigenin-labeled probes according to the method described in Boehringer Mannhein’s Genius System User Guide for Filter Hybridization. In particular, alcohol use status was categorized into nondrinker, moderate alcohol use, and heavy alcohol use through the criteria on daily alcohol consumption, which was defined by Ratten et al. [13], while the uncategorized alcohol consumption, i.e. average alcoholic beverage per day was adjusted in the analyses as a continuous covariate instead of the status to avoid the bias caused by classification. Except for the alcohol use status, methods of coding confounders are described in detail in the Data files, coding manuals, and frequencies for the National Health and Nutrition Survey, available at www.cdc.gov/Nchs/Nhanes, where acquisition processes on the variables and covariates are available.

### Statistical analysis

All statistical analyses were carried out using the R package (www.R-project.org) and EmpowerStats software. In Table 1, the Kolmogorov‒Smirnov test was used to verify the normality of the distribution of continuous variables. Normality values were described as means with standard deviations and compared between the positive and negative groups using one-way ANOVA. With regard to abnormal ones, they were reported as median with interquartile range and compared using the independent-samples Kruskal‒Wallis test. Categorical variables were described as numbers with frequencies and compared using the chi-square test.

Folate in RBCs was stratified into quartiles: Q1 (70–203 ng/ml), Q2 (204–257 ng/ml), Q3 (258–328 ng/ml) and Q4 (329–1022 ng/ml). With the objective of determining the independent association between RBC folate and *T. vaginalis* infection and whether the correlation differed by BMI and age was analyzed by multivariate logistic regression and subgroup analysis stratified by BMI and age. Meanwhile, three models were introduced to enhance the credibility: Unadjusted model, Model I (covariates including age, PIR (Poverty Income Ratio), BMI (Body Mass Index), race, dietary calcium intake and alcohol consumption were adjusted), and Model II (covariates including age, PIR, BMI, race, dietary calcium intake, serum or plasma cholesterol, educational status, marital status and alcohol consumption were adjusted). Odds ratio of *Trichomonas vaginalis* infection was deemed as the major endpoint, which was calculated via the comparison between the infected and the uninfected groups, reflecting the various profiles of the infection rate along with the changing of the exposure. To refrain from arbitrary categorization, smooth fitting curves and generalized additive models were conducted for both overall regression analyses and subgroup analyses, validating the association between continuous overall RBC folate and *T. vaginalis* infection ratio. All P values were calculated, and we regarded P < 0.05 as the criterion to indicate statistical significance.

## Results

### Characteristics of enrolled participants

The baseline characteristics of 1274 participants subclassified by *T. vaginalis* infection status are shown in Table 1, among which, by analyzing the P value, specific characteristics that showed significant differences in the prevalence of the disease were summarized, including RBC folate, BMI, age, PIR, marital status, education status and alcohol consumption (P < 0.05 for each). On average, participants were 33.93 years of age and 27.87 kg/m^2^ for BMI, Compared with the uninfected group, the infected ones tended to be lower income, less calcium intake, more labile marital status, less educational, and consume more alcoholic beverages. Sex was excluded from the table because the participants stretched from the dataset from 2001 to 2004 were all women.

### Association between RBC folate and *Trichomonas vaginalis* infection

To indicate the association between RBC folate and *T. vaginalis* infection, multivariate regression analyses and subgroup analyses stratified by BMI and age were performed. The results are shown in Tables 2 and 3.

**Table 2:** Associations between the quartiles of RBC folate and *T. vaginalis* infection among women

	Unadjusted model		Model I		Model II	
	OR (95% CI)	P value	OR (95% CI)	P value	OR (95% CI)	P value
RBC folate	0.99 (0.99, 1.00)	< 0.0001	0.99 (0.99, 1.00)	0.0003	0.99 (0.99, 1.00)	0.0019
RBC folate quantiles						
Q1 (70–203 ng/ml)	1.0 [Reference]		1.0 [Reference]		1.0 [Reference]	
Q2 (204-257 ng/ml)	0.56 (0.30, 1.02)	0.0007	0.65 (0.34, 1.24)	0.1942	0.68 (0.36, 1.28)	0.2289
Q3 (258-328 ng/ml)	0.15 (0.06, 0.38)	< 0.0001	0.19 (0.07, 0.52)	0.0011	0.20 (0.07, 0.55)	0.0018
Q4 (329-1022 ng/ml)	0.24 (0.11, 0.53)	0.0004	0.30 (0.13, 0.72)	0.0063	0.39 (0.17, 0.92)	0.0305

Unadjusted model: no covariates were adjusted

Model I: Age, PIR, BMI, race, dietary calcium intake and alcohol consumption were adjusted

Mode II: Age, PIR, BMI, race, dietary calcium intake, serum or plasma cholesterol, alcohol consumption, education status and marital status were adjusted

**Table 3:** Subgroup analyses of the association between categorized RBC folate and *T. vaginalis* infection by age and BMI

Subgroup	Participants	RBC folate concentration			
		Q1 (70–203 ng/ml)	Q2 (204–257 ng/ml)	Q3 (258–328 ng/ml)	Q4 (329–1022 ng/ml)
Age					
≤ 35 years	724	1.0 [Reference]	0.54 (0.19, 1.51) 0.2389	0.42 (0.12, 1.45) 0.1689	0.39 (0.09, 1.62) 0.1926
> 35 years	550	1.0 [Reference]	0.74 (0.31, 1.72) 0.4789	0.05 (0.01, 0.44) 0.0068	0.31 (0.10, 0.93) 0.0363
BMI					
≤ 26.505 kg/m^2^	637	1.0 [Reference]	0.72 (0.20, 2.62) 0.6158	0.15 (0.01, 1.46) 0.1018	0.42 (0.06, 2.87) 0.3728
> 26.505 kg/m^2^	637	1.0 [Reference]	0.64 (0.30, 1.39) 0.2635	0.20 (0.06, 0.64) 0.0065	0.30 (0.11, 0.83) 0.0199

All logistic regression models were adjusted for potential confounders [age (excluded in analysis stratified by age), PIR, BMI (excluded in analysis stratified by BMI), race, dietary calcium intake, serum or plasma cholesterol, alcohol consumption, educational status and marital status]

**Fig. 2:**
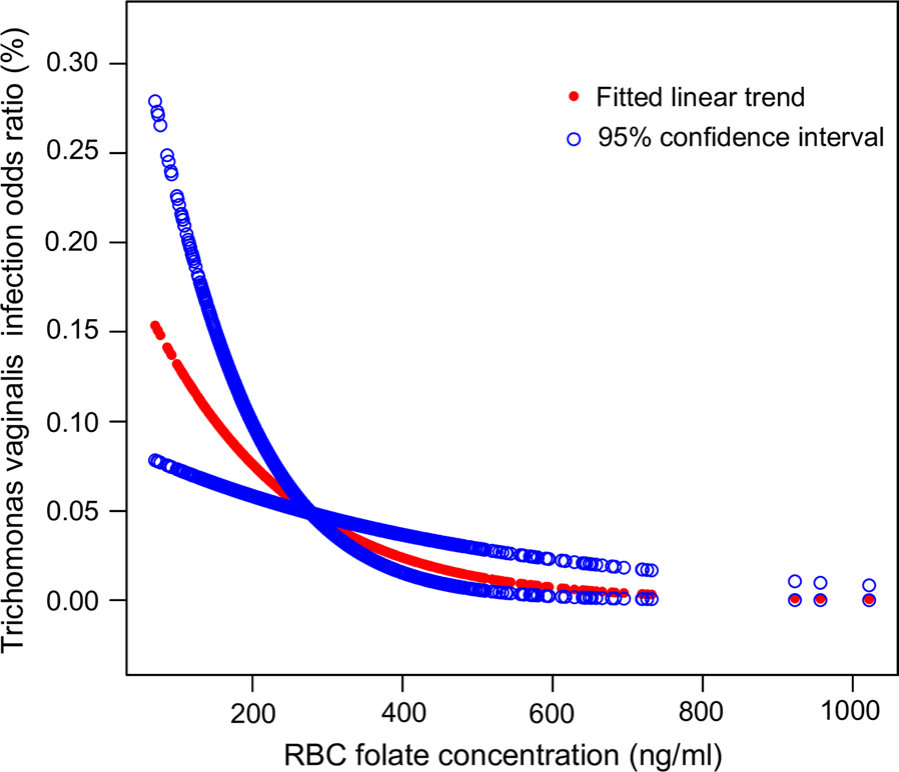
Correlation between RBC folate and *T. vaginalis* infection. The natural spline curve indicates the linear association between RBC folate and the *T. vaginalis* infection ratio (P_linearity_ = 0.0007). The area between the dotted blue lines is regarded as a 95% confidential interval. Each red point reveals the concentration of RBC folate, forming a continuous fitting curve. Ratios are based on model II in multivariate logistic regression models, with all confounders (age, PIR, BMI, race, dietary calcium intake, serum or plasma cholesterol, education status, alcohol consumption and marital status) adjusted

Judging from the multivariate regression analyses, we found a weak correlation between the exposure and outcome variables whether the confounders were adjusted or not (Unadjusted: OR = 0.99 (95% CI 0.99, 1.00) P value < 0.0001, Model I: OR = 0.99 (95% CI 0.99, 1.00) P value = 0.0003, Model II: OR = 0.99 (95% CI 0.99, 1.00) P value = 0.0019). RBC folic acid was converted into quartiles: Q1 (70–203 ng/ml), Q2 (204–257 ng/ml), Q3 (258–328 ng/ml), and Q4 (329–1022 ng/ml). A significantly lower odds ratio of *T. vaginalis* infection was found in Q3 and Q4 in all confounding variables adjusted model (Model II) when compared with Q1, the reference quantile, which reveals a significant negative association between RBC folate and *T. vaginalis* infection (Q2: OR = 0.68 (95% CI 0.36, 1.28), P value = 0.2289, Q3: OR = 0.20 (95% CI 0.07, 0.55) P value = 0.0018, Q4: OR = 0.39 (95% CI 0.17, 0.92) P value = 0.0305). As shown in Fig. 2, smooth curve fitting and generalized additive models focusing on overall RBC folate and *T. vaginalis* infection ratio confirmed the linear negative association (P_linearity_ = 0.0007). Judging from the graph, a more significant negative correlation was found in participants with relatively lower RBC folic acid concentrations. With increasing RBC folate concentration, the negative relevance became weaker.

**Fig. 3:**
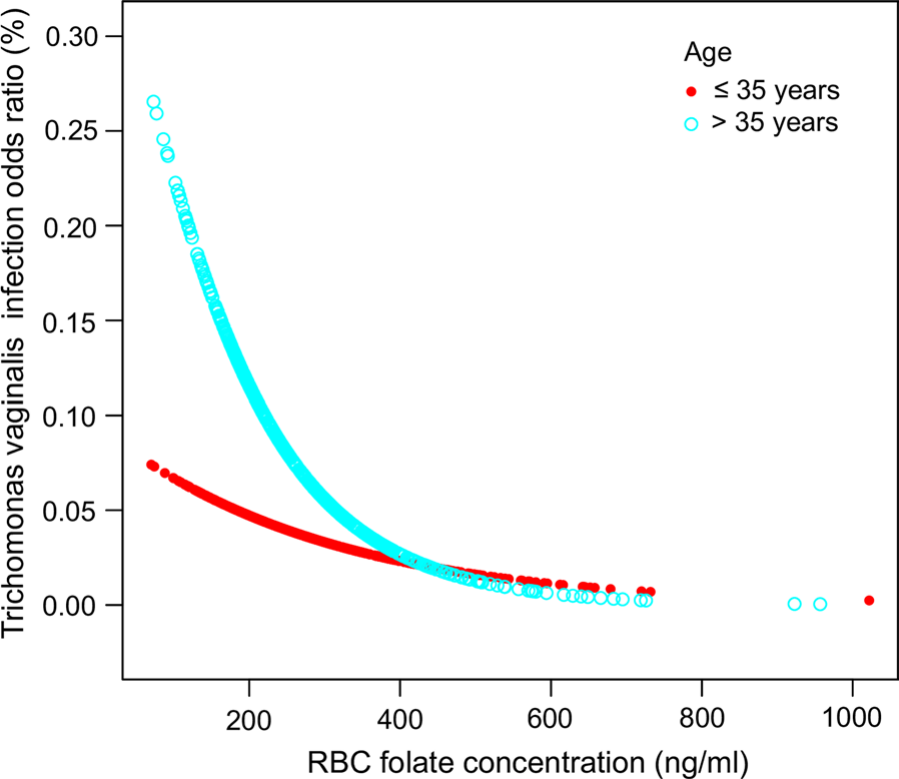
Correlation between RBC folate and *T. vaginalis* infection stratified by age. Fitting curve of the association between RBC folate and *T. vaginalis* infection, stratified by age, with age = 35 taken as the dividing criterion. Age, PIR, BMI, race, dietary calcium intake, serum or plasma cholesterol, education status, alcohol consumption and marital status were adjusted

**Fig. 4:**
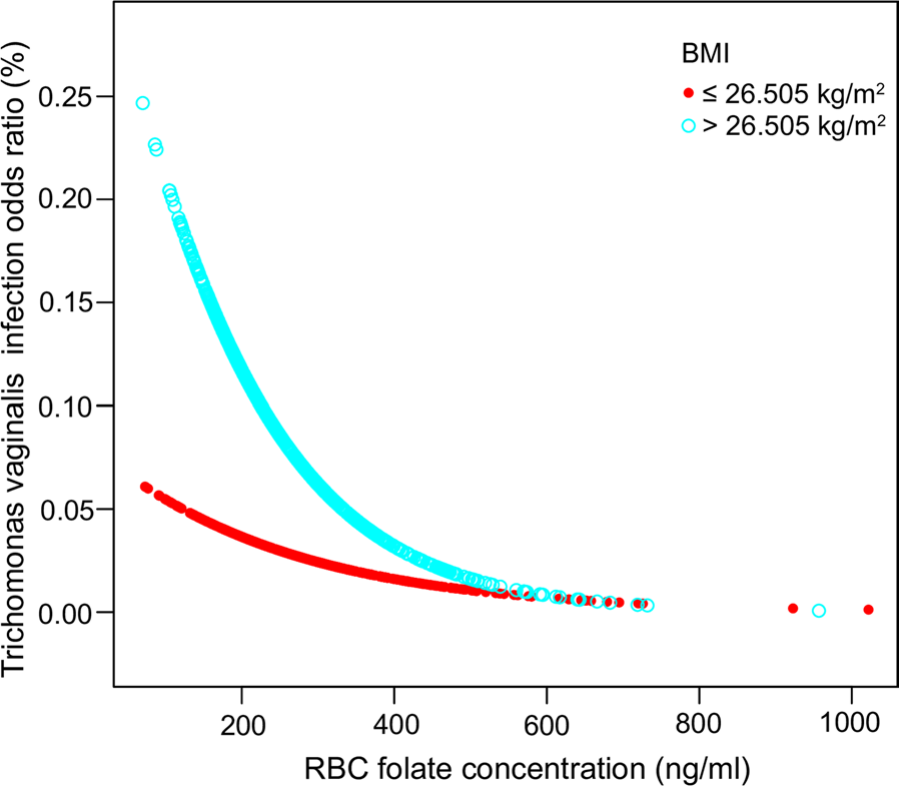
Correlation between RBC folate and *T. vaginalis* infection stratified by BMI. The fitting curve of the association between RBC folate and *T. vaginalis* infection, stratified by BMI, with BMI = 26.505 taken as the dividing criterion. Age, PIR, BMI, race, dietary calcium intake, serum or plasma cholesterol, education status, alcohol consumption and marital status were adjusted

Subgroup analyses and smooth curve fitting models stratified by age and BMI were conducted to further address the modifying effect of potential confounders. Reference as Q1, significant negative correlations between RBC folate and *T. vaginalis* infection were also observed Q3 and Q4 in elder group (age > 35 years old, Q2: OR = 0.74 (95% CI 0.31, 1.72) P value = 0.4789, Q3: OR = 0.05 (95% CI 0.01, 0.44) P value = 0.0068, Q4: OR = 0.31 (95% CI 0.10, 0.93) P value = 0.0363) and high BMI group (BMI > 26.505 kg/m^2^, Q2: OR = 0.64 (95% CI 0.30, 1.39) P value = 0.2635, Q3: OR = 0.20 (95% CI 0.06, 0.64) P value = 0.0065, Q4: OR = 0.30 (95% CI 0.11, 0.83) P value = 0.0199), while weak evidence sustains a significant correlation in youngsters group (age ≤ 35) and low BMI group (BMI ≤ 26.505 kg/m^2^), as detailed in Table 3. Intuitive graphs of curve fittings are shown in Figs. 3 and 4, in accordance with the subgroup results with RBC folate in quartiles (P_linearity for age ≤ 35_ = 0.1929, P_linearity for age > 35_ = 0.0039, P_linearity for BMI ≤ 26.505_ = 0.1689 and P_linearity for BMI ≤ 26.505_ = 0.0022).

## Discussion

In the cross-sectional analysis of two merged datasets from 2001 to 2004 NHANES surveys, our study found a significant negative correlation between RBC folate concentrations and *Trichomonas vaginalis* infection, based on the outcomes of multivariate regression models, with folic acid converted. Subgroup analyses stratified by potential confounders were conducted to better meet the STROBE statement requirements [14]. It was observed that the increase of RBC folic acid concentration was related to the decrease of the possibility of parasitism with *Trichomoniasis vaginalis*. Recently, Naderi, et al. systematically demonstrated that supplementary folate is nutritionally essential for the support of optimal human health and development [15]. In previous studies, low folate was associated with impaired T cells, and folate deficiency was associated with an increased risk of bacteriuria in pregnancy [16, 17]. Nevertheless, it can be challenging to further verify our conclusions with previous analyses because few studies have focused on the latent association between RBC folate and *T. vaginalis* infection. Based on the in vivo folate status of 308 female participants, Janet et al. demonstrated that folate concentration is significantly and independently associated with HR-HPV infection [18]. Another cross-sectional study also showed a synergistic effect of low serum folate with HR-HPV infection in the process of cervical carcinogenesis [19]. Other published studies explored the association between folic acid and gynecological diseases^20^–^24^ . It ought to be noted that there exist studies supporting folate work as a protective factor in *B. vaginosis*, which is another kind of vaginosis. One population-based study found that increased dietary folate may reduce the risk of severe *B. vaginosis* [25], while another study examining women enrolled in the Nashville Birth Cohort (2003–2006) reported a significant atrichossociation between folate deficiencies and *B. vaginosis* [26]. Thus, addressing health problems of women’s folate status may provide the rationale to decrease the infectious ratio of *Trichomonas vaginalis* infection, the homogeneous vaginosis.

The research analyzed representative data from the NHANES database, which employed standardized protocols. According to the current research status, our study is a pioneering observational study examining the relationship between RBC folic acid and *Trichomonas vaginalis* infection. This also has limitations. First, our study is observational and cross-sectional. We cannot validate whether higher RBC folate levels influence changes in *Trichomonas vaginalis* over time. The casual relationship between them is not clear. Therefore, carefully prospective studies with large samples are needed to verify the concrete mechanism of the association between RBC folate concentration and *T. vaginalis* Additionally, due to the possibility of causing the gastrointestinal side effects of anti-parasite drugs, it is potential that participants may alter their B-vitamin status through nutritional supplements [27]. Third, there was concern that there were also male patients infected with *T .vaginalis*, and more complete data are demanded to broaden the study [28]. Last, we did not adjust for other variables, and the participants in the dataset were limited to 2001–2004 due to the subsequent circles recorded by NHANES did not include *T .vaginalis* infection status, the independent variable. Therefore, other potential confounding factors and biases caused by the passage of time cannot be excluded.

## Conclusions

The authors demonstrated a negative association between RBC folate and *Trichomonas vaginalis* infection among representative women of the US. Meanwhile, the benefits of supplementary folate intake might be more significantly reported in older adults and ones equipped with higher body mass indexes. Apart from the general treatment for patients infected with *Trichomonas vaginalis*, a high-folic diet can be recommended additionally by clinicians (e.g. ascended proportion of green leafy vegetables, fruits, and bean products).

## Supplementary Information

**Additional file 1.** Raw data of all recruited participants from NHANES 2001–2004. Data stretched from NHANES 2001–2004 datasets, containing all the needed variables for subsequent analyses from the participants recruited.

**Additional file 2.** Filtered data of the participants enrolled in the study. Data after excluding missing or uninterpreted data from the raw data for variables required for subsequent analyses.

## Data Availability

The datasets generated and analyzed are included in Additional files. The raw data and the data filtered are shared in Additional files 1 and 2, respectively.

## Abbreviations

RBC: Red Blood Cell
*T. vaginalis*: *Trichomonas vaginalis*
NHANES: National Health and Nutrition Examination Survey
IQR: Interquartile range
SD: Standard deviation
PIR: Poverty Income Ratio
BMI: Body Mass Index
CI: Confidence interval
*B. vaginosis*: Bacterial vaginosis
